# Relapsing Polychondritis with Meningoencephalitis Refractory to Immunosuppressant Therapy

**DOI:** 10.1155/2018/1873582

**Published:** 2018-10-03

**Authors:** Mohammad Mousbah Al-Tabbaa, Hani Habal

**Affiliations:** ^1^Visiting MD, University of Illinois College of Medicine at Peoria, Peoria, IL, USA; ^2^Clinical Assistant Professor, University of Illinois College of Medicine at Peoria, Peoria, IL, USA

## Abstract

Meningoencephalitis is a rare complication of relapsing polychondritis. We report a case of a 25-year-old male who presented with visual hallucinations and symptoms of depression and anxiety, white matter changes on MRI, and CSF lymphocytosis, along with inflammatory chondritis seen in his auricle cartilage biopsy. Eventually he was given the diagnosis of RP presenting with meningoencephalitis based on CSF analysis, brain MRI findings, negative serologies, and neurologic exam findings. The patient's clinical state did not improve despite being on IV methylprednisolone for a period of 7 days; afterwards he was switched to oral prednisone with no clinical improvement. As a result, he was given cyclophosphamide and rituximab, respectively, without benefit. He also underwent craniectomy with VP shunt due to worsening hydrocephalus and a brain biopsy was done to confirm the diagnosis. He is currently on methotrexate and steroid dependent with a goal to taper down. Even though all 19 reported cases of meningoencephalitis with RP in the literature did respond to immunosuppressive therapy, in our case, however the patient did not respond to immunosuppressive treatment and currently is in mute dementia status after three years of treatment.

## 1. Introduction

RP is a rare connective tissue disease in which recurrent bouts of inflammation involve the cartilage of the ears, nose, larynx, tracheobronchial tree, and cardiovascular system [1]. Involvement of the peripheral or central nervous system occurs in 3% of patients, sometimes in relation to concomitant vasculitis. Palsies of the cranial nerves (V and VII) are the most common neurological manifestations. Hemiplegia, ataxia, myelitis, and polyneuropathy have been reported. More rarely, aseptic meningitis, meningoencephalitis, stroke, focal or generalized seizures, and cerebral aneurysms may develop [2]. All of the 19 reported cases of meningoencephalitis secondary to RP in the literature did respond to immunosuppressants and most patients age ranges between the 4th and the 5th decade. Here we report a case of a young 25-year-old Caucasian male with a diagnosis of meningoencephalitis secondary to RP that did not respond to different kinds of high potent immunosuppressants. Ultimately, he is being treated palliatively.

## 2. Case Report

**Figure 1:**
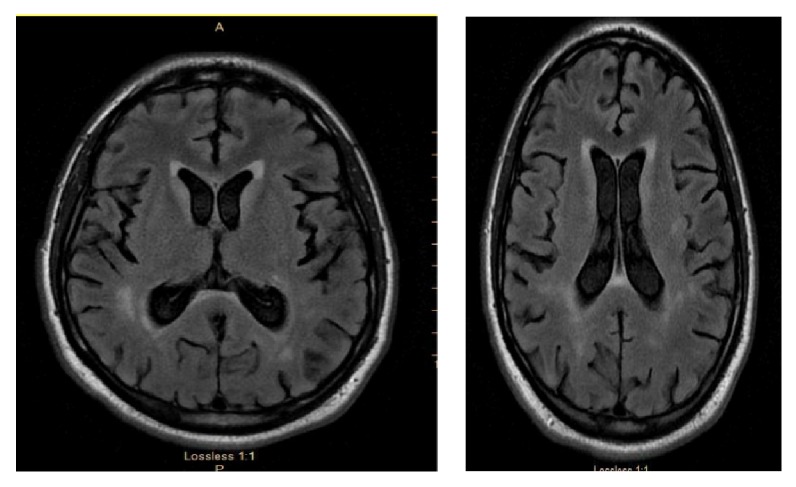
Initial MRI showed diffuse, patchy foci of increased FLAIR signal in the periventricular, deep, and subcortical white matter.

This is a case of a 25-year-old Caucasian male who presented to the ED of St. Francis medical center on 12/2015 with visual hallucinations and symptoms of depression and anxiety, bilateral ear warmth, and swelling and eye redness. His behavioral symptoms have been going for six months prior to presentation. His initial brain MRI showed diffuse, patchy foci of increased FLAIR signal in the periventricular, deep, and subcortical white matter (Figure 1). Right ear lobe biopsy was done and showed a mixed inflammatory infiltrate of the perichondrium composed of plasma cells, lymphocytes, histiocytes and neutrophils, and loss of the cartilage basophilia. GMS and AFB were negative for fungal and mycobacterial organisms. Those findings were consistent with RP.

CSF analysis on admission showed lymphocytosis (21 WBCs, 81% lymphocytes) and admission labs showed lymphocytosis and mildly elevated inflammatory markers (Table 1).

Based on his neurological presentation, his ear lobe biopsy finding, brain MRI findings [3], and negative serologies, he was given a diagnosis of RP with meningoencephalitis.

He was started on IV 1-gram methylprednisolone for 7 days starting in 12/3/15 and then switched to oral prednisone 60 mg/day with a goal to taper off gradually.

The patient's clinical condition did not improve and repeat brain MRI did not show any significant interval change in white matter foci. As a result, the patient was given intravenous cyclophosphamide 1000 mg for total of 5 doses (first 3 doses 3 weeks apart, and another 2 doses 2 weeks apart) between 1/14/2016 and 3/17/2016.

**Figure 2:**
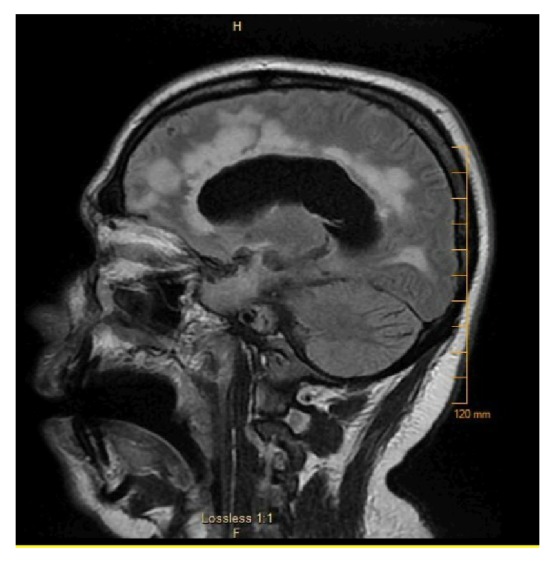
Post-cyclophosphamide MRI showed progressive periventricular, mid, and also a component of superficial/juxtacortical white matter T2/FLAIR hyperintensity, the latter of which is more apparent within the frontal lobes. There has been further progression of hydrocephalus with diffuse ventricular enlargement.

Unfortunately, subsequent MRI after cyclophosphamide on 4/2016 showed progressive periventricular, mid, and also a component of superficial/juxtacortical white matter T2/FLAIR hyperintensity, the latter of which is more apparent within the frontal lobes. There has been further progression of hydrocephalus with diffuse ventricular enlargement (Figure 2).

He was admitted in 5/2016 at SFMC for status epilepticus. Head CT done in the ED showed worsening hydrocephalus. VP shunt was placed and right frontal brain biopsy was done and showed infiltration of the dura and leptomeninges by a mixed chronic inflammatory infiltrate consisting of primarily histiocytes, but also lymphocytes and a few plasma cells. The brain parenchyma shows diffuse gliosis and scant perivascular infiltrates comprised of histiocytes and lymphocytes. No granulomas or vasculitis was identified. Special stains for fungal (GMS and PAS), acid fast (AFB), and bacterial (Gram) organisms are negative. Immunostains for HSV-1/2, CMV, and EBV are also negative. No parasitic organisms are seen on H&E or any of the special stains either. While these morphologic features are nonspecific, they could be consistent with CNS involvement by the patient's known RP. Repeat CSF analysis on the same admission showed 180 RBC, 468 nucleated cells (68% neutrophils, 17% monocytes, and 15% lymphocytes), and normal glucose and protein.

The patient was continued on oral prednisone treatment. A repeat MRI on 12/5/2016 did not show any improvement, so the decision was made to start rituximab and he got 2 doses in January 2017.

**Figure 3:**
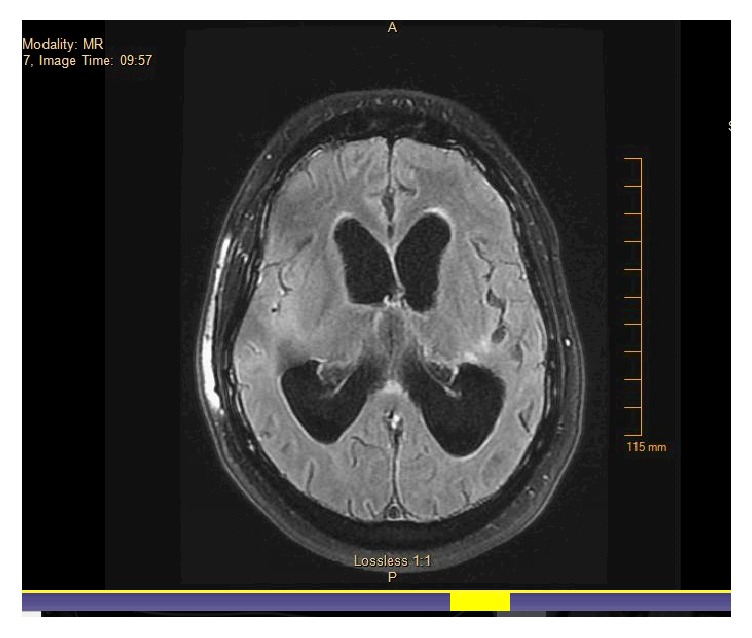
Post-rituximab MRI did not show any significant interval change in findings suggesting leptomeningitis/pachymeningitis, and foci of prolonged T2 values within the white matter.

Subsequent brain MRI on 5/26/17 did not show any significant interval change in findings suggesting leptomeningitis/pachymeningitis, and foci of prolonged T2 values within the white matter (Figure 3).

Currently the patient is in stable mute dementia status. He is alert. Language skills are very limited (up to one or two words mostly). He is relatively distractible. He can follow commands, but this is usually when demonstrated to him. He is currently on prednisone 15 mg/day which is being tapered off, and he is on methotrexate 25 mg/week since 4/2018 which was added as a steroid sparing agent to help taper down the prednisone dose.

## 3. Discussion

Neurologic complications of RP are rare in general; in the literature limbic encephalitis was reported and the patient presented with cognitive dysfunction [4]. Another case reported rhombencephalitis in which the patient presented with severe occipital headache and this resulted in brain edema and death in two months [5]. Most of the reported cases in the literature reported vasculitic encephalitis, limbic encephalitis, and rarely aseptic meningoencephalitis.

Neurologic presentation of meningoencephalitis with RP varied between cases; reported presentations were hearing loss, unsteadiness, personality changes, generalized tonic-clonic seizure, impaired cognitive function, impaired visual acuity, confusion, memory loss, unstable gait, ataxia, hydrocephalus, delirium, cerebral infarction, coordination problems, distraction, word finding difficulty, emotional lability, abducens nerve palsy, anxiety, insomnia, memory loss, deafness, gait change, urinary incontinence, expressive and receptive aphasia, dullness, acalculia, and papilledema [6].

In the literature all cases of RP-related meningoencephalitis were in the older patient population than our patient and had responded well to immunosuppressive therapy. There are no current guidelines for meningoencephalitis due to RP, because of its rarity. Corticosteroids were the main therapy along with other immunosuppressants. The choice of immunosuppressant varies in the literature, although azathioprine and cyclophosphamide were most commonly used. The choice of immunosuppressant was empirical, with no specific immunosuppressant reported for meningoencephalitis due to RP, due to the lack of clinical trials and cohorts for this specific complication.

Treatment trends differed between cases. 10 of 19 cases improved on glucocorticoids and 5 out of those 10 showed a relapse of disease. Cyclophosphamide was added to glucocorticoids in 3 cases, one case died [7], one improved with no relapse [8, 9], and the most recent case had to add cyclosporin A to relieve symptoms, even though the patient relapsed [6]. Azathioprine was added to glucocorticoids in 4 cases all of which showed improvement, but three of those cases relapsed [6].

Currently, there are no guidelines for treatment of meningoencephalitis in RP; all the reported cases responded to immunosuppressive therapy with glucocorticoids alone or with other high potent immunosuppressants like cyclophosphamide and azathioprine. Our case did not show any improvement to two high potent immunosuppressive therapies (cyclophosphamide and rituximab). Currently he has irreversible dementia and is dependable on steroids with the goal of tapering off his prednisone as a palliative measure.

## 4. Conclusion

Although meningoencephalitis due to RP usually responds to immunosuppresive therapy, our case is an example of a severe case of RP-related meningoencephalitis that is refractory to any treatment regimen.

## Figures and Tables

**Table 1:** Labs on admission.

ESR	22 mm/hr (ref. 0-15)

Urine drug screen	negative

CRP	1.76 mg/dl (ref. less than 0.50)

CSF, cell count	RBCs: 7 Nucleated cells: 21

CSF, differential	Neutrophils: 3% Lymphocytes: 81% Monocytes: 16%

CSF, VDRL	Negative

CSF, autoimmune encephalopathy panel	Negative

CSF, Lactic acid	WNL

Paraneoplastic panel	Negative

HIV 1&2 antibody	Negative

ASO	Negative

AFP	Negative

RPR	Non-reactive

ANA	Negative

ENA panel	Negative

VIT b12	WNL

Cryoglobulin	Negative

Hepatitis B and C	Negative

ANCA MPO PR3	Negative

C3&C4	WNL

SPEP (check)	No abnormal protein band detected

WNL: within normal limit, SPEP: serum protein electrophoresis, ref: reference.

## Abbreviations

RP: Relapsing polychondritis
MRI: Magnetic resonance imaging
ED: Emergency department
VP: Ventriculoperitoneal.
